# Non-invasive Assessment of Rheumatoid Arthritis Cardiac Involvement: A Systematic Review of Echocardiography

**DOI:** 10.2174/0115734056375528250701165335

**Published:** 2025-07-11

**Authors:** Huang Xingxing, Chen Tianyi, Yu Xiaolong

**Affiliations:** 1 Wujin Hospital Affiliated with Jiangsu University, Changzhou, Jiangsu, China; 2 The Wujin Clinical College of Xuzhou Medical University, Changzhou, Jiangsu, China

**Keywords:** Rheumatoid arthritis, Echocardiography, Speckle tracking echocardiography, The pressure-strain loop, Autoimmune diseases, Mortality rates, Heart

## Abstract

**Background:**

Rheumatoid arthritis (RA) is a systemic autoimmune disorder primarily characterized by joint degradation, with consequential cardiovascular ramifications significantly impacting patient mortality rates.

**Methods:**

We systematically searched for full-text English-language journal articles from 1973 to 2025 in the PubMed and Web of Science databases. Utilizing keywords such as “Rheumatoid Arthritis,” “Autoimmune Diseases,” “Pathophysiology,” “Heart,” “Cardiac,” and “Echocardiography” to narrow the search results. Articles related to the evaluation of heart diseases in rheumatoid arthritis by echocardiography were included, while those with insufficient data or low data quality were excluded. Study quality was assessed using the CASP Quantitative Checklist (2018 version), and data were synthesized through thematic content analysis.

**Results:**

We included 52 studies in this review after the primary analysis. The results show that traditional echocardiography can identify organic changes in the heart and ventricular function impairment of patients with rheumatoid arthritis. New ultrasound techniques, such as speckle tracking and pressure-strain loops, can detect ventricular function impairment earlier than traditional echocardiography.

**Discussion:**

Echocardiography provides complementary diagnostic information for rheumatoid arthritis cardiac involvement through structural and functional assessment, yet limitations remain. Future work should establish multimodal ultrasound frameworks and develop AI-driven analytical platforms to enhance early detection and precision management.

**Conclusion:**

The continuous progress of ultrasound technology has significantly improved the accuracy of assessing cardiac damage in patients with rheumatoid arthritis, and it has become an essential examination method for screening heart diseases in such patients, providing strong support for early diagnosis.

## 1 INTRODUCTION

RA is a commonly encountered autoimmune disease in clinical practice, with a global prevalence of approximately 1%. It disproportionately affects women, who are about three times more likely to be affected than men [1]. The primary pathological features of RA include synovitis and joint bone destruction, accompanied by multi-organ damage. Of these, cardiovascular system involvement is particularly significant, encompassing pericardial disease, valvular heart disease, cardiomyopathy, coronary artery disease, and arrhythmias. Studies indicate that early mortality in RA patients is primarily due to cardiovascular diseases (CVD) [2], especially ischemic heart disease and congestive heart failure (HF) [3, 4]. Even with conventional treatment for ischemic heart disease, the risk of HF in RA patients is double that of non-RA individuals, and the risk of death due to HF is also twice as high [5]. However, early cardiac damage in RA patients is often subclinical and easily overlooked. By the time symptoms become evident, the cardiac damage is often severe and irreversible. Therefore, early detection and management of cardiac damage in RA are crucial.

In the evaluation of cardiac involvement in patients with rheumatic diseases, echocardiography serves as an effective non-invasive modality [6]. Owing to its convenience and high reproducibility, echocardiography has emerged as an optimal choice for assessing cardiac damage in RA patients. Advancements in ultrasonography, including novel diagnostic techniques like speckle tracking and stress-strain loop analysis, have significantly augmented the diagnostic potential of echocardiography. These advancements facilitate the detection of early subclinical cardiac involvement.

This article reviews the historical development of ultrasound assessment for cardiac damage in RA, comprehensively discusses the research progress of echocardiography in the non-invasive assessment of cardiac damage in RA, and looks forward to the potential of this technology in the clinical evaluation of RA cardiac damage. It aims to provide clinicians with a new perspective on managing patients' cardiac health.

## 2 MATERIALS AND METHODS

To review the progress of studies on the use of echocardiography in assessing cardiac damage in patients with RA, we systematically searched for related reviews, cohort studies, and case-control studies published from 1973 to 2025 in the PubMed and Web of Science databases. Utilizing keywords such as “Rheumatoid Arthritis,” “RA,” “Autoimmune Diseases,” “Heart,” “Cardiac,” “Cardiovascular,” “Speckle tracking,” “Pressure strain loop,” “Doppler,” “Three-Dimensional,” and “Echocardiography,” and employing “AND” and “OR” to broaden or narrow the search result, we conducted preliminary screenings.

Studies were included if they met all of the following criteria:

(a) Population: Enrolled adult patients (≥18 years) with diagnosed RA and confirmed cardiac involvement, (b) Intervention: Employed echocardiography for cardiac evaluation, (c) Outcomes: Reported quantitative or qualitative echocardiographic parameters related to RA cardiac assessment, (d) Study design: Original research articles (cohort, case-control, or cross-sectional studies with ≥20 participants)

Studies were excluded for any of the following:

(a) Publication type: Reviews, editorials, conference abstracts, theses, (b) Irrelevance: Did not focus on echocardiographic evaluation of RA-associated cardiac disease, (c) Data limitations: Insufficient methodological details or Incomplete outcome data, (d) Language: Non-English publications, (e)Duplication: Overlapping datasets without novel analyses.

The study evaluation was independently completed by two blinded reviewers. During the literature screening process, the titles and abstracts of the articles were read first. After excluding the studies that were obviously irrelevant, two reviewers were assigned to conduct independent evaluations of each included study by means of the CASP Quantitative Checklist. In the event that there are discrepancies in the evaluation results obtained by the two reviewers, both of them are required to conduct a secondary review of the corresponding literature to ensure that the literature finally included in the study possesses a high quality.

**Fig. (1):**
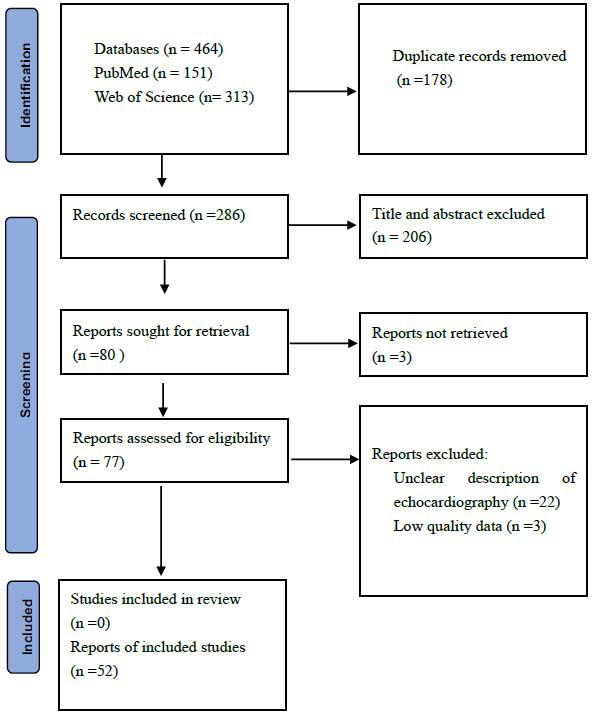
PRISMA literature selection flowchart.

This study employed the content analysis method to analyze the relevant studies on the evaluation of cardiac involvement in RA using echocardiography. The specific text segments in the studies regarding the application of ultrasonic technology for the evaluation of cardiac involvement in RA were determined as the analysis units. A detailed coding system was constructed focusing on the pathophysiological mechanisms of cardiac involvement in RA, the types of echocardiographic techniques, such as traditional echocardiography, speckle tracking echocardiography, the pressure-strain loop, as well as the commonly used evaluation indicators for disease diagnosis, assessment of disease progression, and prognosis judgment. Two researchers were assigned to independently carry out the coding work. After the coding was completed, the data were statistically analyzed. Then, with the help of the cross-analysis method, the application differences of different echocardiographic techniques in diagnosing various types of cardiac involvement in RA were explored, so as to reveal the application status of echocardiography in the evaluation of cardiac involvement in rheumatoid arthritis. Literature selection followed PRISMA guidelines, as shown in the PRISMA flowchart (Fig. **1**).

## 3 RESULTS

Through systematic retrieval in the PubMed, Embase, and Web of Science databases, 464 potentially relevant articles were identified with specific keywords. After removing 178 duplicate pieces of literature, 286 remained for the next screening step. Subsequently, based on the comparison of titles and abstracts, 206 pieces of literature with weak relevance were excluded, and 80 articles were reserved for in-depth reading. Through careful reading, it was found that 22 of them did not focus on the key points of the evaluation of echocardiography for the cardiovascular system in rheumatoid arthritis, and another 3 pieces of literature had low-quality data. A total of 52 relevant articles were ultimately included in this study.

### 3.1 Rheumatoid Arthritis Cardiac Involvement Pathophysiology

#### 3.1.1 Coronary Artery Disease

Although the precise relationship between RA and coronary artery disease has not been fully elucidated, chronic inflammation, autoimmune responses, and hematological abnormalities are believed to play crucial roles [7]. A study involving 418 patients with acute myocardial infarction revealed an independent association between serum levels of Rheumatoid Factor (RF) and the complexity of coronary artery lesions (assessed *via* SYNTAX score I) [8]. Additionally, Hannawi *et al*. found that the thickness of the left anterior descending artery wall in RF-positive RA patients was more significant compared to RF-negative patients [9], suggesting a correlation between RF presence and coronary artery disease in RA patients. The median serum concentrations of inflammatory cytokines such as Interleukin-6 (IL-6), Tumor Necrosis Factor-alpha (TNF-α), and myeloperoxidase are significantly elevated, which can induce endothelial dysfunction, insulin resistance, dyslipidemia, and aberrant activation of the coagulation cascade, thereby accelerating atherosclerosis [10]. Endothelial dysfunction can also promote the production of Reactive Oxygen Species (ROS), affecting vasomotor function and leading to microvascular dysfunction. In addition, RA can lead to abnormal activation of the immune system and the production of numerous cytokines [11], among which T cells and natural killer (NK) cells can promote vasoconstriction through the renin-angiotensin system while inhibiting the vasodilatory effects of adenosine and acetylcholine, resulting in microvascular dysfunction [12].

#### 3.1.2 Heart Failure

HF is one of the most severe complications of RA. A study encompassing 905 RA patients found that the cumulative incidence rate of HF events in the RA cohort was approximately 7.67%-12.64% [13]. Inflammation is considered the most critical mechanism for the occurrence of HF. It has been proposed that myocardial cells respond to chronic inflammation by producing cytokines and adhesion molecules, increasing leukocyte recruitment within the cardiac cells and reducing myocardial contractility [14]. Additionally, research by Lim *et al*. discovered that chronic inflammation leads to microvascular and endothelial dysfunction, resulting in myocardial remodeling and fibrosis, ultimately causing left ventricular (LV) systolic dysfunction (LVSD) [15]. Notably, LV diastolic dysfunction (LVDD) is more common in RA patients and is considered a significant pathogenic pathway to HF, although it may remain asymptomatic for an extended period [16, 17]. Studies have found that increased oxidative stress in RA patients leads to reduced bioavailability of nitric oxide (NO) and decreased levels of cyclic guanosine monophosphate (cGMP) and protein kinase G (PKG) in the myocardium, further promoting cardiac hypertrophy and increased resting tension [18, 19]. Moreover, macrophages with high expression of Major Histocompatibility Complex class II (MHC II) produce osteopontin and Transforming Growth Factor-beta (TGF-β) in response to autocrine stimulation by IL-10, leading to fibroblast proliferation, increased collagen deposition, and consequent cardiac stiffness and LVDD [20]. A 4-6-year follow-up study of 158 RA patients without clinical HF found that IL-6 is associated with LV concentric remodeling and can serve as a biomarker for ventricular remodeling in RA patients without clinical HF [21]. The use of tumor necrosis factor-alpha (TNF-α) inhibitors in the treatment of RA patients reduces the risk of HF [22].

#### 3.1.3 Arrhythmias

Patients with RA have an increased risk of arrhythmias compared to non-RA populations [23], with atrial fibrillation being the most common type of arrhythmia observed [24]. Inflammatory cytokines in RA can promote abnormal proliferation of cardiac fibroblasts by altering the expression ratio of matrix metalloproteinases (MMPs) to their tissue inhibitors (TIMPs), further driving fibroblast differentiation into myofibroblasts and leading to excessive collagen deposition. This fibrosis reduces ventricular conduction velocity, decreases repolarization reserve, promotes early afterdepolarizations, and generates reentry circuits, ultimately leading to arrhythmias in patients with RA [25]. Additionally, inflammatory cytokines in RA can directly alter transmembrane ion currents, reduce the expression of connexins in cardiomyocytes, and alter their distribution, thereby changing the electrophysiology of the myocardium [26-28]. Certain antibodies found in RA patients, particularly anti-Ro antibodies, have been associated with arrhythmias; they bind to L-type calcium channels and Kv11.1 channels, increasing arrhythmia risk [29]. Furthermore, research indicates that the prevalence of atrial fibrillation in female RA patients is higher than in males, suggesting that estrogen may influence atrial fibrillation [30]. However, some studies argue there is no link between atrial fibrillation and RA, attributing differences to patient selection and treatment variations [31].

#### 3.1.4 Pericardial Disease

The pericardium is one of the most commonly affected cardiac structures in patients with RA. RA patients ' autoantibodies and circulating immune complexes may deposit in the pericardial tissue, triggering inflammatory responses and tissue damage. Studies have linked pericardial involvement to elevated inflammatory cytokine levels (such as IL-1, IL-6, and TNF-α) in RA patients [32, 33]. Additionally, increased activated T cells in RA patients may further activate macrophages and other inflammatory mediators, thereby inducing pericarditis. Clinically, pericardial involvement may manifest as pericardial thickening, pericardial effusion, or nodules. The most common clinical presentation is asymptomatic pericardial effusion, with the volume of effusion typically being moderate. The production of these effusions may be due to fluid exudation caused by pericardial inflammation, hemodynamic abnormalities, or fluid leakage due to low colloid osmotic pressure [32].

#### 3.1.5 Valvular Heart Disease

Valvular lesions in patients with RA are generally thought to be associated with systemic chronic inflammation response in which inflammation-induced proliferation and death of the associated cells trigger cardiac remodeling, but some scholars have also suggested that a polygenic, antigen-driven response plays a significant role in the pathogenesis of RA-associated valvular heart disease [34]. Immune complex deposits are frequently observed in the annulus and base of the valve in patients with RA, and they may also be found in the central portion of the valve. These lesions are typically characterized by the presence of well-defined, single or multiple, ovoid nodules at the central margins of the valve leaflets. Among the rheumatoid valvular lesions, mitral and aortic insufficiency are the most common ultrasound manifestations [35, 36].

### 3.2 The Application of Echocardiography in Cardiac Damage in RA Patients

Echocardiography is widely applied in the assessment of cardiac damage in RA patients, providing an accurate evaluation of the type and extent of cardiac damage and serving as a clinical guide post-medication. Compared to other imaging tests like CT and MRI, echocardiography offers several advantages, including being non-invasive, convenient, cost-effective, having strong repeatability, and allowing real-time dynamic monitoring. With the continuous advancement of related technologies, echocardiography has developed various types, leading to an increasingly profound understanding of cardiac involvement in RA patients. In 1973, literature first reported the mitral valve and pericardium involvement in RA patients using two-dimensional echocardiography [37]. Davia and others reported in 1975 that mitral valve motion abnormalities in RA patients could be identified through Trans-Thoracic Echocardiography (TTE) [38]. Later studies found that TTE could identify pericardial effusion, ventricular function impairment, and DD [39, 40], along with the application of Speckle tracking echocardiography (STE) in the diagnosis of early myocardial dysfunction [41] and the introduction of the concept of non-invasive myocardial work based on the Pressure-strain loop (PSL) [42].

**Fig. (2):**
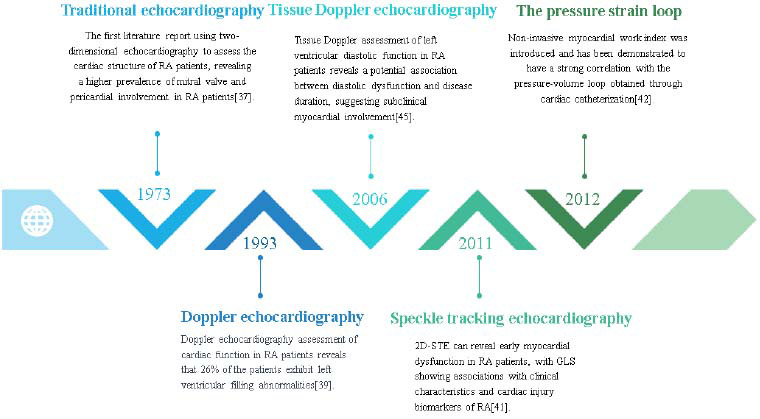
Developmental history of the application of echocardiography in the assessment of cardiac damage in rheumatoid arthritis.

These findings are crucial for understanding the impact of RA on the heart. We have reviewed the historical application of echocardiography in assessing cardiac damage in RA (Fig. **2**) and summarized the clinical application of different echocardiographic techniques in the evaluation of cardiac damage in RA.

#### 3.2.1 Traditional Echocardiography

##### 3.2.1.1 Two-dimensional Echocardiography and M-mode Echocardiography

Two-dimensional echocardiography and M-mode echocardiography represent the predominant techniques utilized in cardiac ultrasound examinations, offering dynamic visualizations and intricate structural details essential for evaluating cardiac damage associated with RA. These technologies allow for the direct observation of cardiac morphology and functional abnormalities. They facilitate the detection of changes in heart size, ventricular remodeling, pericardial and valvular alterations, as well as abnormalities in wall motion, thereby offering crucial information for the early detection and treatment of cardiac diseases. In 1973, Dr. Ravi Prakash and colleagues conducted a pioneering study utilizing two-dimensional echocardiography to evaluate asymptomatic RA patients. Their findings revealed cardiac abnormalities in a significant proportion of subjects: 44% exhibited pericardial effusion, while 25% demonstrated reduced E-F slope of the anterior mitral leaflet. This prospective clinical study provided the first *in vivo* evidence of mitral valve and pericardial involvement in RA patients, phenomena that had previously only been documented in postmortem examinations [37]. Subsequent research by D. Schorn *et al*. on patients with active RA revealed a high overall incidence of cardiac involvement of 73% [43]. Similarly, studies by S. Corrao and colleagues have identified at least three typical alterations in RA patients without any cardiac symptoms: posterior pericardial effusion, changes in the aortic root, and valvular thickening [44]. Severe pericardial effusion is relatively rare in RA patients; however, its occurrence typically accompanies other serious symptoms such as constrictive pericarditis or cardiac tamponade. Research has demonstrated that ultrasound-guided pericardiocentesis can safely and effectively treat this rare complication in RA patients [45]. Valve involvement is also common in RA patients. Guedes *et al*. used Transesophageal Echocardiography (TEE) and found valvular involvement in 83% of RA patients, most commonly presenting as valvular thickening and nodules. In contrast, the detection rate of valvular involvement through TTE is only about 53% [46]. In summary, echocardiographic abnormalities in the pericardium are common in RA patients but are typically clinically insignificant, and a decrease in the E-F slope detectable by two-dimensional echocardiography is rarely observed in RA patients [38, 47, 48].

Studies have demonstrated that patients with RA can exhibit cardiac eccentric remodeling prior to the manifestation of clinical features associated with CVD. Research conducted by Valeria *et al*. identified that the relative wall thickness (RWT), an easily obtainable echocardiographic parameter, and the reduction of RWT can be utilized to assess early cardiac damage in RA [49]. An echocardiographic study involving 89 RA patients without clinical cardiovascular disease found an association between RA and increased LV mass, independently correlated with the presence of LV hypertrophy (odds ratio 4.14 [95% confidence interval 1.24, 13.^80^]). This suggests a pathophysiological link between chronic inflammation in RA and LV hypertrophy [50]. Cioffi *et al*. conducted M-mode echocardiographic measurements to evaluate aortic elastic properties in patients with RA. The study demonstrated significantly higher ascending aortic stiffness index (AoSI) values in RA patients compared with non-RA controls (p<0.001). Notably, elevated AoSI levels were established as a reliable predictor of adverse clinical outcomes during mid-term follow-up in the RA cohort [51]. High AoSI is also believed to be associated with clinically increased LV mass and subclinical LV dysfunction in RA [52].

##### 3.2.1.2 Doppler Echocardiography

Left heart dysfunction in RA patients may be related to disease duration and severity [53]. Within this context, the most common manifestation is LVDD. Commonly used parameters for assessing LVDD include peak velocities of early (E) and late (A) diastolic blood flow across the mitral valve, the E/A ratio, diastolic (D wave) and systolic (S wave) blood flow velocities in the pulmonary veins, the S/D ratio, and isovolumetric relaxation time (IVRT). LVDD often precedes systolic dysfunction, and studies have shown that RA patients with preserved LV ejection fraction (LVEF) are more prone to LVDD compared to non-RA individuals. Abnormalities in the E/A and S/D ratios may indicate subclinical LVDD [54], while changes in mitral valve hemodynamics reflect subclinical myocardial involvement related to the disease course [55]. Further research has established a significant correlation between the severity of RA disease activity and LVDD(p < 0.0001) [56]. Studies using Tissue Doppler Imaging (TDI) have revealed significantly reduced S', E,' and E'/A' ratios at the basal septum and lateral annulus of the mitral valve in patients with early-stage RA, indicating preclinical myocardial impairment [57]. The research data from Seyfeli and colleagues demonstrate that TDI exhibits significantly higher sensitivity (89%) than conventional echocardiography (34%) in detecting right ventricular diastolic dysfunction among RA patients (*p*=0.002) [58]. This finding underscores the diagnostic superiority of TDI in cardiac functional assessment for this patient population. Funda Levendoglu and colleagues employed Doppler echocardiography to assess cardiac function in patients with active RA. Their investigation revealed bilateral ventricular diastolic dysfunction, demonstrating significant correlations between disease duration and: (1) the ratio of E to A ventricular filling velocities (E/A ratio: r=-0.47, *p*=0.002), and (2) isovolumetric relaxation time (IRT: r=0.618, p<0.001). These findings suggest a direct relationship between left ventricular diastolic function parameters and RA disease progression [59]. Giovanni Cioffi and others demonstrated that approximately 45% of RA patients exhibited reduced tissue Doppler S' values, indicating subclinical systolic dysfunction independent of LVEF. This impairment showed significant correlations with diastolic function parameters (E/E' ratio) and blood pressure load. Notably, the distribution of S' impairment remained consistent across all LV geometric patterns (*p*=0.14), suggesting that RA-related myocardial injury may preferentially affect longitudinal fiber mechanics rather than inducing global ventricular remodeling [60]. Studies by Alpaslan and colleagues have shown that early LVDD in RA patients can be assessed using the Myocardial Performance Index (MPI) and mitral valve blood flow propagation velocity (TFPV) beyond common echocardiographic parameters. MPI, derived from pulsed Doppler measurements of ventricular inflow and outflow velocities, reflects overall ventricular function and is less affected by local wall motion abnormalities. TFPV, obtained from color Doppler M-mode echocardiography, is useful for evaluating LV diastolic function. Both parameters are easily obtainable and less influenced by geometric changes [40]. Moreover, Doppler echocardiography has also been proven effective in assessing cardiac function in RA patients following pharmacological treatment [61].

#### 3.2.2 Speckle Tracking Echocardiography

STE operates by frame-by-frame tracking of the geometric movement of uniformly distributed speckles within the myocardium in two-dimensional grayscale images. This is achieved through spatial and temporal image processing algorithms to glean tissue motion information, thereby obtaining multiple motion parameters in different directions [62]. STE is utilized to measure myocardial strain in various directions and is clinically employed to assess myocardial dysfunction. Previous research has demonstrated that the global longitudinal strain (GLS) measured by STE is more sensitive than standard echocardiographic parameters. It overcomes the limitations of the LVEF and can detect cardiac dysfunction not identifiable by standard echocardiography. Currently, it is considered the most accurate and sensitive parameter for assessing early LV dysfunction [41, 63, 64].

Many studies have found that the LV longitudinal strain and right ventricular strain in RA patients were lower than those in non-RA individuals [65, 66]. Buakhamsri *et al*. demonstrated that RA exhibited significantly reduced GLS values (-2.8%, *p*=0.03) despite having preserved LVEF and normal diastolic function parameters. This finding suggests the presence of subclinical myocardial impairment in this patient population. Furthermore, the study identified significant correlations between GLS impairment and both RA disease duration (*p*=0.02) and disease activity (DAS28-CRP, *p*=0.041), implying that chronic inflammatory processes may contribute to myocardial fibrosis or microvascular dysfunction. However, these conclusions should be interpreted with caution due to the study's limited sample size (n=59), necessitating validation in larger cohorts [67]. A prospective STE study involving 209 RA patients without overt cardiac disease revealed that while isolated reductions in either GLS or global circumferential strain (GCS) showed no significant prognostic value, the concurrent presence of both abnormal GLS and GCS independently predicted cardiovascular events. Importantly, this combined strain impairment remained significantly associated with adverse outcomes after adjustment for aortic stiffness. However, the median 16-month follow-up limits long-term interpretation, warranting extended observation [68].

Naseem *et al*. demonstrated significant correlations between rheumatoid arthritis disease activity (assessed by both SDAI and DAS28 scores) and impaired biventricular myocardial function, as evidenced by reduced GLS values (all p<0.001). Their receiver operating characteristic analysis revealed excellent predictive accuracy of these disease activity indices for subclinical cardiac dysfunction, with area under the curve values exceeding 0.90 for both ventricular assessments. Based on these robust associations, the authors proposed that RA patients with SDAI scores >7 or DAS28 scores >2.8 should undergo comprehensive cardiac evaluation to facilitate early identification of high-risk individuals [69]. Fine *et al.* documented significantly reduced biventricular strain in RA patients (LV: -15.7% *vs* -18.1%; RV: -17.9% *vs* -20.7%, both p<0.001), independently associated with HAQ-DI scores (*p*=0.032). These findings support incorporating strain imaging into cardiovascular risk stratification for RA, especially in long-standing or highly inflammatory disease [70]. A study conducted in Denmark found that RA patients with persistently elevated anti-CCP antibodies demonstrated significant deterioration in myocardial function over a 2-year period, as evidenced by reduced S' velocity (*p*=0.04) and impaired global longitudinal strain (GLS, *p*=0.04). Notably, the degree of GLS impairment showed a direct correlation with anti-CCP levels (r=0.36, *p*=0.006). These findings support the potential role of anti-CCP as a biomarker for myocardial injury and highlight the clinical value of speckle-tracking echocardiography in detecting subclinical cardiac dysfunction in RA patients. However, the statistical power of these observations may be limited by the relatively small sample size in the high anti-CCP subgroup [71]. An animal experiment, to some extent, explained this phenomenon, showing that high inflammation in collagen-induced arthritis (CIA) rats is associated with impaired LV diastolic function and greater myocardial deformation unrelated to hemodynamic load [72]. However, a recent meta-analysis on speckle tracking technology presented a different view. They found that compared to non-RA patients, RA patients had significantly reduced GLS and GCS, but there was no statistically significant difference in right ventricular GLS. Additionally, there was no association between GLS and disease duration, activity or inflammatory markers [73], which might be attributed to the loss of individual patient characteristics in the combined analysis.

Several studies have focused on the relationship between the disease course of RA and the assessment parameters of 2D-STE. Baktir *et al*. revealed that STE exhibits significantly higher sensitivity than conventional echocardiography in detecting early signs of subclinical myocardial damage (including abnormalities in radial and longitudinal strain parameters) among RA patients with disease duration exceeding 10 years [74]. Ji and others revealed a strong negative correlation between peak strain dispersion (PSD) and LVGLS (r=-0.91, P<0.01), with PSD increasing significantly with RA disease duration (r=0.85, P<0.01). These findings suggest that myocardial mechanical dispersion may reflect chronic RA-associated electromechanical coupling abnormalities. Notably, the lack of significant association between PSD and inflammatory markers indicates that these changes are likely driven by non-inflammatory myocardial remodeling processes, such as fibrosis [75].

Three-dimensional STE (3D-STE) is more sensitive in evaluating cardiac motion than 2D-STE, as it overcomes the spatial limitations of two-dimensional planes, providing a comprehensive quantification of myocardial mechanics in a single heartbeat. It introduces new strain parameters, such as global area strain (GAS), which combines longitudinal and circumferential strain characteristics to reflect changes in the endocardial surface area throughout a cardiac cycle. This means that 3D-STE can precisely analyze LV geometric structures and displacements, identifying echocardiographic patterns of various diseases with similar phenotypes [76]. Feng *et al*. demonstrated that while conventional echocardiographic parameters remained within normal ranges in RA patients, 3D-STE revealed significantly impaired multidirectional myocardial strain parameters (GLS, GRS, GCS, and GAS; all P<0.05). These findings highlight the superior sensitivity of 3D-STI in detecting subclinical left ventricular systolic dysfunction in RA patients, providing an objective basis for early clinical intervention [77]. A comparative study of two-dimensional speckle tracking technology and real-time three-dimensional echocardiography evaluating LV contraction synchrony in patients with RA showed that both 2D-STI and RT-3DE could detect changes in LV synchrony in RA patients and quantitatively evaluate LV contraction synchrony. However, RT-3DE is more sensitive in detecting changes in LV synchrony [78].

Many studies suggest that changes in GLS can accurately assess cardiac function changes in RA patients after medication [79, 80]. Research by Yasemin Ozden Ayyildiz and others found that long-term inhibition of TNF-α with infliximab in patients with RA improves LV longitudinal and radial arterial systolic deformation and reduces LV torsion (LVtor) [74]. Another similar study assessing cardiac function in RA patients with STE found that a three-month treatment with infliximab improved left atrial (LA) abnormalities [81]. Konomidis and colleagues observed impaired baseline myocardial deformation indices in RA patients related to nitrosative stress and endothelial dysfunction [82]. Inhibiting interleukin-1 activity improved nitrosative stress and endothelial and coronary artery function, thereby improving LV deformation.

#### 3.2.3 The Pressure-strain Loop

The PSL technique combines brachial systolic blood pressure (SBP) with GLS, which is measured by 2D-STE, to calculate the myocardial work index (MWI) [83]. The most significant advantage of this method lies in its capacity to evaluate the impact of loading conditions on LV deformation without necessitating left heart catheterization. It mitigates load dependency and exhibits a strong correlation with pressure-volume loops derived from cardiac catheterization, rendering it a viable tool for assessing early cardiac functional impairment [84]. The principle behind PSL is to integrate afterload measurements with layered strain technology to overcome load dependency and to show a good correlation with catheter-based pressure-volume loops, making it suitable for evaluating early cardiac functional impairment [85-87]. MWI represents the total work done by the myocardium from the closure to the opening of the mitral valve within a cardiac cycle. Changes in MWI levels can reflect alterations in global and regional LV mechanics under different loading conditions. Key parameters include the global myocardial work index (GWI), global myocardial constructive work (GCW), global myocardial wasted work (GWW), and global myocardial work efficiency (GWE).

Liu and colleagues' research demonstrates that in patients with RA, the absolute values of GLS, LVGWI, LVGCW, and LVGWE gradually decrease with the progression of the disease, while LVGWW increases over time. Among PSL parameters, LVGWW has the highest capability to identify cardiac function impairment in RA patients. LVGCW is significantly reduced in groups with longer disease duration, primarily due to more severe myocardial damage and more apparent LVSD in patients with longer disease courses. In groups with shorter disease duration, an increase in LVGWW and a decrease in LVGWE were observed, while the absolute value of GLS showed no significant difference compared to the control group, indicating that PSL parameters are more sensitive than GLS in detecting LVEF [88]. Yu *et al.* revealed significantly impaired myocardial work parameters (GWI, GCW, and GWE; all P<0.05) in patients with high disease activity, demonstrating a dose-dependent relationship between inflammatory burden and mechanical cardiac dysfunction [89]. However, studies by Ren *et al*. and Xiao *et al*. showed a good correlation between RA patients' GWI, GCW, and LVEF, LVGLS, demonstrated the feasibility of LV PSL technology in assessing cardiac function in RA patients, and found no statistically significant difference in GWW and GWE compared to control groups [90, 91]. This difference may be due to the different criteria for selecting research characteristics in different studies. A study revealed significant sex-specific differences in myocardial work parameters: female participants exhibited a progressive age-related decline in global work efficiency (GWE), while males demonstrated only linear increases in constructive work (GCW). Notably, patients in the high-risk Framingham category showed markedly elevated rates of abnormal wasted work (GWW) and impaired work efficiency (GWE), both reaching 11% prevalence (P<0.001) [92].

## 4 DISCUSSION

In the chapter on traditional echocardiography, a total of 26 research findings were included. These studies focused on traditional echocardiography. Among them, the literature regarding two-dimensional echocardiography and M-mode echocardiography mainly conducted research on the organic heart diseases of patients with RA. The research content not only covers morphological and structural abnormalities such as valve involvement, pericardial diseases, and myocardial lesions, but also includes the evaluation of left ventricular systolic dysfunction. The studies related to Doppler echocardiography, on the other hand, focused on the assessment of diastolic dysfunction in RA patients, and indicated that diastolic dysfunction occurs earlier than systolic dysfunction in the disease process. Traditional echocardiography is the most widely applied technique for clinical assessment of cardiac damage in patients with RA, encompassing M-mode echocardiography, two-dimensional echocardiography, conventional Doppler echocardiography, and TDI. The simplicity of operation and high reproducibility of traditional echocardiography make it especially valuable. M-mode echocardiography, characterized by its high temporal resolution, is adept at depicting cardiac motion, making it suitable for evaluating the movement of cardiac valves. However, the significant limitation of M-mode echocardiography in clinical application is its insufficient spatial resolution. Two-dimensional echocardiography provides high-resolution structural information about the heart, clearly delineating the thickness of cardiac walls, the interventricular septum, and cardiac valves while also allowing for the real-time observation of cardiac motion and contraction. Nonetheless, two-dimensional echocardiography has certain limitations in assessing the velocity and direction of cardiac motion, particularly in evaluating LV diastolic function. Doppler echocardiography, which does not require puncture or contrast injection, can present real-time cardiac blood flow images, measuring the velocity and direction of cardiac blood flow and providing information on cardiac hemodynamics to assist in evaluating cardiac function and disease status. However, Doppler echocardiography's sensitivity to the angle of blood flow necessitates the correct angulation to avoid measurement errors and ensure the accuracy of blood flow velocity measurements. TDI offers a detailed assessment of cardiac tissues with higher temporal and spatial resolution. Despite TDI's complexity and limited application range, it excels in providing in-depth insights into the condition of cardiac tissues. Utilizing various types of echocardiography comprehensively provides extensive cardiac evaluation data, offering significant support for the early diagnosis and treatment of cardiac involvement in RA patients.

In the chapter on STE, a total of 20 research findings were included. These studies focused on the application of STE in the assessment of myocardial dysfunction in patients with RA. The study found that the parameters of STE can detect cardiac insufficiency that is difficult to identify by traditional echocardiography. On this basis, some studies have further explored the correlations between STE parameters and the disease course as well as the disease activity of RA. STE, as an emerging echocardiographic technique, has been fully validated. Compared with LVEF determined by traditional echocardiography, GLS measured by STE is more sensitive in the assessment of myocardial dysfunction. Clinical research data show that, compared with LVEF, GLS exhibits more significant prognostic value in predicting major adverse cardiac events in patients with RA, providing strong support for the implementation of personalized risk assessment for RA patients. With this characteristic, GLS not only helps clinicians formulate more targeted risk prevention and control strategies but also becomes a valuable tool in scientific research and clinical practice. However, currently, the relationship between GLS and the disease activity and course of RA has not yet reached a consensus. There are differences in the conclusions of existing studies. It is urgent to conduct large-scale, multicenter studies to further verify the accuracy and reliability of GLS in the assessment of cardiac involvement in RA, so as to promote the standardized application of this technique in the diagnosis and treatment of cardiac lesions in RA.

In the chapter on PSL, a total of 6 research findings were included. These studies focused on the parameters of PSL and conducted an in-depth exploration of the correlations between these parameters and the disease course and disease activity of patients with RA. However, these studies have not reached a consensus in this field, and there are certain differences in the research results. PSL is an effective non-invasive method for assessing myocardial energetics, offering broad prospects in diagnosis and prognosis. It has been applied in evaluating various types of cardiac diseases, including coronary artery disease, hypertension, cardiac amyloidosis, hypertrophic cardiomyopathy, and dilated cardiomyopathy [93]. This method provides a more comprehensive assessment of overall cardiac function and performance and has been proven to have better sensitivity and specificity in assessing left heart function compared to traditional echocardiography [94]. GCW and GWW, as measures of cardiac reserve and energy loss, respectively, also provide additional information to support commonly used echocardiographic parameters in clinical practice [95]. The novel non-invasive LV PSL area measurement method aligns well with invasive measurements and direct myocardial work assessment, reflecting myocardial metabolic conditions [42]. This is an advanced echocardiographic technique capable of early cardiac function impairment assessment and may have clinical significance for various patient groups, including cardiac evaluation in RA patients, showcasing its potential in the medical field. However, current studies in the RA population are extremely limited in sample size, raising debates over the validity and accuracy of the assessments. Future researchers are encouraged to conduct more extensive studies with larger samples to validate the feasibility and accuracy of PSL in assessing cardiac involvement in RA patients, providing a more comprehensive parameter for the non-invasive assessment of cardiac function in RA patients.

Despite significant progress in echocardiographic technology, each type of echocardiography still has distinct advantages and disadvantages (Table **1**). Additionally, we are confronted with the challenges posed by the complexity of the disease and the diversity of patients. RA exhibits a wide range of clinical heterogeneity, leading to varied types and degrees of cardiac damage.

The future development trends in echocardiography may focus on the following aspects: Firstly, the integration of multimodal ultrasound imaging, which combines ultrasound with other imaging technologies, such as CT and MRI, is currently a hot research topic. This integration is expected to provide more comprehensive and accurate diagnostic information. Secondly, the application of Artificial Intelligence (AI) in the interpretation of echocardiograms will undoubtedly enhance the automation level of echocardiographic technology [96], reduce dependence on the operator's skill, and effectively improve efficiency and accuracy through AI-assisted diagnosis. This will lead to the standardization of measurements, assist doctors in diagnosing heart diseases, optimize clinical workflows, and ultimately reduce healthcare costs [97]. Thirdly, the application of functional ultrasound imaging in cardiac assessment, including speckle tracking technology and non-invasive myocardial work, has already entered clinical practice, with the potential for developing more advanced functional imaging technologies to provide more comprehensive information beyond structural imaging. Fourthly, the development of ultrafast ultrasound imaging technologies is currently limited by traditional frame rates in echocardiography. Compared to traditional imaging, ultrafast ultrasound imaging can capture images at frame rates up to 100 times faster [98]. Specific applications of this technology have been developed and tested for clinical use in pediatric and adult cardiac imaging, including ultrafast Doppler or vector flow imaging, shear wave imaging, electromechanical wave imaging, and backscatter tensor imaging [98]. Fifthly, the organic integration of molecular imaging with echocardiography is still in progress. In biological systems, visualizing, describing, and measuring biological processes at the molecular and cellular level through the chemical synthesis of molecular probes, nanomaterials, and other agents targeting specific proteins or genes represent a promising biomedical approach. The future of imaging lies in molecular imaging combined with specific targeting agents [99-101].

## CONCLUSION

With continuous advancements in medical technology, echocardiography has become crucial in diagnosing and managing CVD in patients with RA. Technological improvements have significantly enhanced its accuracy and reliability in detecting cardiac involvement, enabling early diagnosis, timely intervention, and optimized treatment strategies. These developments pave the way for more precise and personalized cardiac care in RA, ultimately improving clinical outcomes and quality of life.

## Figures and Tables

**Table 1:** Advantages and disadvantages of various types of echocardiography.

**Types of Echocardiography**	**Advantage**	**Disadvantage**
Two-dimensional echocardiography and M-mode echocardiography	A two-dimensional echocardiogram can provide real-time cross-sectional images of the heart's structure, allowing for the observation of the heart's size, shape, and movement of its various parts. M-mode echocardiography provides one-dimensional images of time and motion, enabling precise measurement and recording of the movement of cardiac structures.	With relatively low sensitivity, the accuracy of numerical measurements can be influenced by changes in cardiac structure, while image quality may be affected by factors such as intrathoracic gas and chest wall deformities. It is not particularly sensitive in assessing regional variations in myocardial function and diastolic dysfunction.
Doppler echocardiography	It can be employed to assess the velocity and direction of blood flow as well as the functioning of cardiac valves, aiding in the diagnosis of valve disorders and abnormalities in intracardiac blood flow.	Its accuracy is highly contingent upon the angle between the ultrasound beam and the direction of blood flow. Numerical measurements can be influenced by alterations in cardiac structure, while image quality may be affected by intrathoracic gas and chest wall deformities.
Speckle tracking echocardiography	It can detect subtle myocardial motion and deformation, exhibiting a high sensitivity for assessing myocardial function. It allows for quantitative analysis of myocardial strain in various directions, is angle-independent, and demonstrates strong reproducibility.	It exhibits load dependency, demands a high level of image quality, requires a higher proficiency level from medical practitioners, and lacks uniformity in image and data processing across different vendors.
The pressure-strain loop	It demonstrates high sensitivity for assessing myocardial function, enabling non-invasive evaluation of myocardial work. It is angle-independent, load-independent, and exhibits strong repeatability.	High image quality standards, a high level of proficiency required from the medical practitioners, and inaccurate results in cases where patients have concurrent conditions causing disparities between arterial pressure and left ventricular pressure.

## Data Availability

The data that support the findings of this study are available on request from the corresponding author [Y.X] upon reasonable request.

## LIST OF ABBREVIATIONS

RA: Rheumatoid arthritis
CVD: Cardiovascular diseases
HF: Heart failure
TNF-α: Tumor Necrosis Factor-alpha
IL-6: Interleukin-6
NK: Natural killer
ROS: Reactive Oxygen Species
LVDD: LV diastolic dysfunction
LV: Left ventricular
cGMP: Cyclic guanosine monophosphate
PKG: Protein kinase G
NO: Nitric oxide
MHC II: Major Histocompatibility Complex class II
TGF-β: Transforming Growth Factor-beta
MMPs: Matrix metalloproteinases
PSL: Pressure-strain loop
TTE: Trans-Thoracic Echocardiography
RWT: Relative wall thickness
IVRT: Isovolumetric relaxation time
MPI: Myocardial Performance Index
CIA: Collagen-induced arthritis
GAS: Global area strain
LVtor: LV torsion
AI: Artificial Intelligence

## STANDARDS OF REPORTING

PRISMA guidelines and methodology were followed.
